# Androgen receptors and serum testosterone levels identify different subsets of postmenopausal breast cancers

**DOI:** 10.1186/1471-2407-12-599

**Published:** 2012-12-14

**Authors:** Giorgio Secreto, Elisabetta Venturelli, Elisabetta Meneghini, Maria Luisa Carcangiu, Biagio Paolini, Roberto Agresti, Cristina Pellitteri, Franco Berrino, Massimo Gion, Patrizia Cogliati, Giuseppina Saragò, Andrea Micheli

**Affiliations:** 1 Department of Preventive and Predictive Medicine, Fondazione IRCCS Istituto Nazionale dei Tumori, Milan, Italy; 2 Descriptive Studies and Health Planning Unit, Fondazione IRCCS Istituto Nazionale dei Tumori, Milan, Italy; 3 Department of Pathology, Fondazione IRCCS Istituto Nazionale dei Tumori, Milan, Italy; 4 Breast Surgery Unit, Fondazione IRCCS Istituto Nazionale dei Tumori, Milan, Italy; 5 Association for Application of Biotechnologies in Oncology and Center for the Study of Biological Markers of Malignancy, Regional Hospital, Local Health Unit (AULSS) No 12, Venice, Italy; 6 Scientific Consultant for Scientific Direction and Descriptive Studies and Health Planning Unit, Fondazione IRCCS Istituto Nazionale dei Tumori, Milan, Italy

**Keywords:** Androgen receptors, Androgens, Postmenopausal breast cancer, Testosterone levels

## Abstract

**Background:**

Androgen receptors (AR) are frequently expressed in breast cancers, but their implication in cancer growth is still controversial. In the present study, we further investigated the role of the androgen/AR pathway in breast cancer development.

**Methods:**

AR expression was evaluated by immunochemistry in a cohort of 528 postmenopausal breast cancer patients previously examined for the association of serum testosterone levels with patient and tumor characteristics. AR expression was classified according to the percentage of stained cells: AR-absent (0%) and AR-poorly (1%-30%), AR-moderately (>30%-60%), and AR-highly (>60%) positive.

**Results:**

Statistical analysis was performed in 451 patients who experienced natural menopause. AR-high expression was significantly related with low histologic grade and estrogen receptor (ER)- and progesterone receptor (PR)-positive status (*P* trend<0.001). Mean testosterone levels were significantly higher in the AR-high category than in the other categories combined (*P*=0.022), although a trend across the AR expression categories was not present. When women defined by ER status were analyzed separately, regression analysis in the ER-positive group showed a significant association of high testosterone levels with AR-highly-positive expression (OR 1.86; 95% CI, 1.10-3.16), but the association was essentially due to patients greater than or equal to 65 years (OR 2.42; 95% CI, 1.22-4.82). In ER-positive group, elevated testosterone levels appeared also associated with AR-absent expression, although the small number of patients in this category limited the appearance of significant effects (OR 1.92; 95% CI, 0.73–5.02): the association was present in both age groups (<65 and ≥65 years). In the ER-negative group, elevated testosterone levels were found associated (borderline significance) with AR-absent expression (OR 2.82, 95% CI, 0.98-8.06). In this ER-negative/AR-absent subset of tumors, elevated testosterone levels cannot stimulate cancer growth either directly or after conversion into estrogens, but they probably induce increased synthesis of some other substance that is responsible for cancer growth through binding to its specific receptor.

**Conclusions:**

The findings in the present study confirm that testosterone levels are a marker of hormone-dependent breast cancer and suggest that the contemporary evaluation of ER status, AR expression, and circulating testosterone levels may identify different subsets of cancers whose growth may be influenced by androgens.

## Background

Androgen receptors (AR) are expressed in more than 70% of primary breast cancers [1-11], in up to 90% of estrogen receptor (ER)-positive [5,7-12] and in about 50% of ER-negative tumors [6-8,11]. AR positivity was found associated with favorable tumor characteristics, such as small tumor size, low histological grade, ER- and progesterone receptor (PR)-positive status [5-9,12], and with better outcomes than in patients with AR-negative tumors [1,2,4,6,11,12]. Such findings suggest that AR play a role in breast cancer development, but the clinical-pathological implication of the androgen/AR pathway on cancer growth is not yet well known.

We recently examined the relationships of serum testosterone levels with some tumor characteristics in a cohort of postmenopausal breast cancer patients and found that high levels of testosterone were significantly associated with the ER-positive status of tumors [13,14], a finding suggesting that serum testosterone levels are a marker of hormone-dependent breast cancer.

In the present study, we examined AR expression in tumors of the same cohort of postmenopausal patients in whom we had already measured testosterone levels. With the aim to further investigate the role of the androgen/AR pathway in breast cancer development, we evaluated the relationships of AR expression with testosterone levels, with age and body mass index (BMI) of patients and with selected tumor characteristics, both in the whole cohort and in age categories (<65 and ≥65 years).

## Methods

### Patients

Of the 592 initial postmenopausal breast cancer women recruited in the TPM (testosterone, prognosis, mammary cancer) cohort, for 538 we had information on AR expression. Inclusion and exclusion criteria of the TPM cohort were extensively presented elsewhere [13,14] and are here briefly summarized: all patients with primary breast cancer were treated surgically at the Breast Surgery Unit of the Fondazione IRCSS Istituto Nazionale Tumori, Milan (INT) from December 2003 to December 2006. Inclusion criterium was having histologically confirmed non-metastatic breast carcinoma (any T, any N, M0); exclusion criteria were nonepithelial cancer, a previous cancer diagnosis (except in situ cervical cancer or nonmelanoma skin cancer), and neoadjuvant chemotherapy or hormone therapy. The women provided a fasting blood sample before surgery; the blood samples were processed, divided into aliquots and stored at −80°C. Patient information and hormone receptor status (ER, PR, HER2) were extracted from the clinical records; collected data were entered into a specific database. Written informed consent was obtained from all included patients. The study was approved by the Scientific and Ethics Committee of the INT.

Of the 538 women with information on AR expression, 2 women aged 51–52 were excluded because they were not considered postmenopausal on the basis of their estradiol level (i.e., estradiol in serum was higher than 30 pg/ml). Eight women were excluded because they proved to fall outside the recruitment criteria. Specifically, one was excluded because the date of the last menstruation was less than 1 year before the date of recruitment, and the other seven women were excluded because they had stopped hormone replacement therapy only 3 months before recruitment. Thus, 528 women were available for the present analysis.

Mean age at recruitment was 66.8 years (standard deviation 9.0, range 41–97). Four hundred and fifty-one women were going through natural (self-reported, nonsurgical) menopause (mean age, 66.6±8.8; range, 41–91); of the remaining 77 women, 42 had undergone hysterectomy, 32 had undergone bilateral ovariectomy, and 3, with uncertain self-reported information, were classified as missing status for menopause type. This cross-sectional study is mainly addressed to those 451 naturally menopausal breast cancer patients. Analyses on all (natural and surgical) postmenopausal breast cancer patients are included in the Additional file 1.

### Testosterone assay

Testosterone assay, already described elsewhere [14], was performed using RIA commercial kits (Orion Diagnostica, Espoo, Finland) according to the manufacturer’s instructions. The coefficients of variation of measurement were 6.4% and 7.6% for mean testosterone titers of 0.359 and 0.455 ng/ml, respectively.

### Androgen receptor assay

AR expression was evaluated using tissue microarrays. Slides and paraffin blocks of 538 consecutive invasive breast cancers were retrieved from the archives of the department of pathology of the INT. All slides hematoxylin and eosin stained were reviewed by two pathologists, and the slide with the most representative tumor section was selected for each case. Three representative core tissue samples (1.5 mm in diameter) were taken from different areas of the invasive tumor and an additional one was from benign breast tissue, when present, and were assembled in 45 tissue microarray paraffin blocks using a Galileo TMA CK3500 Tissue Microarrayer. From each tissue microarray block, 4-mm-thick sections were cut and immunostained for monoclonal mouse anti-human androgen receptor, Dako (clone AR441, dilution 1:50) using Dako Autostainer.

### Statistical methods

ER and PR status were defined as negative when the percentage of stained tumor cells was <10% and as positive for ≥10% of stained tumor cells, in accordance with published guidelines [15]. No guidelines have been published to define AR status. In most studies, the cutoff value of 10% of stained cells was used to separate AR-negative from AR-positive tumors [5,8,9,16]. Hu et al. [11] reported three groups of AR expression, negative (0% of stained cells), low positive (1-10%), and positive (>10%), and Ogawa et al. [7] divided their patients in four groups according to AR expression, none, low (<10%), intermediate (10-50%), high (>50%). In order to further analyze the association between AR and testosterone levels, we classified AR expression in four categories: AR-absent (0%), AR-poorly (≥1% to 30%), AR-moderately (>30% to 60%), and AR-highly (>60%) positive. The cutoff of 1% was chosen because the association with testosterone levels became clearly visible, mainly in the ER-negative subset, when tumors with no cells stained for AR were separately considered. The cut offs of 60% and 30% were arbitrarily chosen to distinguish AR-high expression and divide the AR positive expression in groups with approximately equal numbers of patients. We did not further divide patients using the cutoff value of 10%, commonly employed in other studies, because testosterone levels in the category ≥1% to 10% (61 patients, mean testosterone 0.396±0.182) did not differ significantly from those in the category >10% to 30% (50 patients, mean testosterone 0.373±0.194) (p=0.409).

Differences in patient (age and BMI) and tumor characteristics (tumor size, histology, tumor grade, ER status, PR status, HER2 status and axillary involvement) between AR expression categories were investigated by the chi-square test for trend.

Testosterone circulating levels were square-root transformed, as the distribution of concentrations was not normal. Fisher’s test was used to assess overall differences in mean testosterone levels by categories of AR expression, and linear contrasts were used for post-hoc comparisons of the high AR category versus absent, poor and moderate AR combined categories and of the absent AR category versus poor and moderate AR combined categories. In case of multiple comparisons, the Bonferroni method was used to adjust *P* values. Trends across AR expression categories were tested by the nonparametric Cuzick test.

Logistic regression analysis was used to assess the age-adjusted association between testosterone and AR expression, and the age-adjusted odds ratios (OR) of being in a given AR expression category rather than in the reference category were estimated across testosterone tertiles. The ER status resulted as a determinant of the relationship between testosterone and AR expression. In particular, (a) in the ER-positive group, the frequency of women with low AR expression did not differ from that of women with moderate AR expression, thus the poor and moderate AR expression categories were pooled and used as reference category in estimating the age-adjusted OR of developing a tumor with absent or high AR expression versus one with the reference AR expression. (b) In the ER-negative group, there were no differences between low, moderate or high AR expression, so all three categories of positive AR expressions were pooled together and used as reference in a binomial logistic model estimating OR of having AR-absent instead of AR-positive tumors.

Testosterone tertiles were categorized according to the distribution of women in natural menopause. Although the likelihood ratio test did not reveal any significant interaction between age and testosterone, data were separately analyzed in order to take into account the hormonal pattern modification during years after the menopause in women <65 and those ≥65 years of age.

Ninety-five percent confidence intervals (95% CI) were estimated. Linear trends in OR were tested using ordinal variables of testosterone tertiles. All *P* values refer to two-sided statistical tests; differences with *P*≤0.05 were considered significant. Analyses were performed with the Stata statistical package, 9.2 (2007) release (Stata Corporation, College Station, TX, USA).

## Results

Of 528 postmenopausal women, 451 (85.4%) were naturally menopausal and 74 (14.0%) had had a surgical menopause (due to missing data on the type of menopause - natural or surgical, 3 other women were excluded). Compared to naturally menopausal women, those who had had surgical menopause were more frequently in the ≥65 year age class (64.9% vs. 53.7%, *P*=0.072), more frequently had a BMI ≥30 kg/m^2^ (28.8% vs. 17.2%, *P*=0.026), more frequently had an ER-negative tumor (24.3% vs. 15.8%, *P*=0.071), and more frequently had lower mean testosterone levels (0.346±0.176 vs. 0.418±0.196 ng/ml, Fisher’s *P*=0.001). Furthermore, no association between AR expression and testosterone levels was found for women who had had surgical menopause [see Additional file 1, Table S2]. For this reason, we herein present results on naturally menopausal women only, and the results on all postmenopausal women are shown in the additional tables [see Additional file 1].

Table 1 shows frequencies of patients and tumor characteristics within categories of AR expression. About 13% of women in natural menopause had AR-absent tumors, 51% had tumors with poor or moderate AR expression, and 36% of women had tumors with AR-high expression (last row of Table 1). Tumors with high AR expression were slightly more frequent (not significantly) than tumors with lower AR expression in women aged ≥ 65 years (58% vs. 51-53%). Tumors with high AR expression were less frequently associated (not significantly) to the large tumor size — ≥2 cm — than tumors with AR-absent expression (29% vs. 45%). The frequency of infiltrating ductal carcinoma decreased (not significantly) across increasing categories of AR expression (89%, 80%, 81%, and 78%). The frequency of tumors grade ≤2 strongly increased across increasing AR expression (38%, 61%, 62%, and 70%) (*P* trend <0.001), as did the frequency of women with ER-positive or PR-positive tumors: 53%, 82%, 89%, and 94% (*P* for trend <0.001), and 42%, 61%, 69%, and 77% (*P* for trend <0.001), respectively. In the HER2-positive group, the incidence of women with poor and moderate AR expression tended to be higher than that of women with AR-absent or AR-high expression (58% and 52% vs. 41%). In these naturally menopausal women, AR expression did not appear to be related to BMI or nodal status.

**Table 1:** **Androgen receptor** (**AR**) **expression and characteristics of breast cancer women in natural menopause**

	**AR expression**								
	**Absent**		**Poor**		**Moderate**		**High**		
	**No.**	**%**^**b**^	**No.**	**%**^**b**^	**No.**	**%**^**b**^	**No.**	**%**^**b**^	***P***^**c**^
Age, yr									
< 65	29	49.2	55	49.1	57	47.5	68	42.5	0.259
≥ 65	30	50.8	57	50.9	63	52.5	92	57.5	
BMI, kg/m^2^									
< 25	28	50.0	45	46.9	58	52.7	68	47.2	0.478
25-30	18	32.1	41	42.7	34	30.9	44	30.6	
≥ 30	10	17.9	10	10.4	18	16.4	32	22.2	
Tumor size, cm									
< 2	32	55.2	74	66.7	70	58.8	112	70.9	0.080
≥ 2	26	44.8	37	33.3	49	41.2	46	29.1	
Histology									
Infiltrating ductal carcinoma (IDC)	50	89.3	87	79.8	95	80.5	124	77.5	0.110
Other infiltrating carcinoma	6	10.7	22	20.2	23	19.5	36	22.5	
Grade									
≤ 2	22	37.9	68	61.3	72	61.5	112	70.4	<0.001
> 2	36	62.1	43	38.7	45	38.5	47	29.6	
Axillary nodal status									
Negative	34	60.7	66	60.6	74	62.7	95	60.1	0.958
Positive	22	39.3	43	39.4	44	37.3	63	39.9	
ER status									
Negative	28	47.5	20	18.0	13	10.8	10	6.3	<0.001
Positive	31	52.5	91	82.0	107	89.2	149	93.7	
PR status									
Negative	34	57.6	42	38.2	37	30.8	36	22.6	<0.001
Positive	25	42.4	68	61.8	83	69.2	123	77.4	
HER2 status^a^									
Negative	30	58.8	35	41.7	40	48.2	64	59.3	0.332
Positive	21	41.2	49	58.3	43	51.8	44	40.7	
Total	59	13.1 ^d^	112	24.8 ^d^	120	26.6 ^d^	160	35.5 ^d^	

NOTES. Androgen receptor (AR) expression: absent, 0%; poor, ≥1 to 30%; moderate, >30 to 60%; high, >60%. Estrogen receptor (ER)-positive and Progesterone receptor (PR)-positive: ER and PR expression ≥10%.

^a^One hundred and twenty-five (27.7%) women had missing information about HER2 status.

^b^Column percentage. ^c^Chi-square test for trend. ^d^Row percentage.

Mean testosterone levels by categories of AR expression are presented in Table 2. When all women in natural menopause were considered, the AR-high category showed the highest mean testosterone level, which was significantly different from that of the other categories combined (linear contrast: *P*=0.002), although a trend across the AR expression categories was not shown. When women defined by ER status were separately analyzed, those in the ER-negative group showed significantly lower mean testosterone levels than those in the ER-positive group (0.369 ng/ml vs. 0.428 ng/ml, Fisher’s *P*=0.023). In the ER-positive group, women with high AR expression had the highest testosterone level (linear contrast: Bonferroni adjusted *P*=0.022), yet women in the AR-absent category showed high testosterone levels, although not significantly different from those in low and moderate combined AR categories (linear contrast: Bonferroni adjusted *P*=0.216). In the ER-negative group, women with AR-positive expressions had lower, not significantly, testosterone levels than women in the AR-absent category.

**Table 2:** Serum testosterone by androgen and estrogen receptor expression in natural postmenopausal women with breast cancer

	**Testosterone**			
	**No**.	**%**	**Mean ± SD (ng/ml)**	***P***
All natural menopause				
AR expression				
Absent	59	13.1	0.434 ± 0.198	0.099^b^ 0.002^c^ 0.002^d^
Poor	111	24.7	0.385 ± 0.187	
Moderate	120	26.8	0.384 ± 0.166	
High	159	35.4	0.462 ± 0.215	
Total^a^	449	100.0	0.419 ± 0.196	
ER-positive				
AR expression				
Absent	31	8.2	0.451 ± 0.203	0.116^b^ 0.003^c^ 0.022^d,f^ 0.216^e,f^
Poor	91	24.1	0.402 ± 0.189	
Moderate	107	28.3	0.385 ± 0.170	
High	149	39.4	0.470 ± 0.217	
Total	378	100.0	0.428 ± 0.200^g^	
ER-negative				
AR expression				
Absent	28	39.4	0.415 ± 0.193	0.510b 0.204^c^
Poor	20	28.2	0.310 ± 0.160	
Moderate	13	18.3	0.378 ± 0.124	
High	10	14.1	0.345 ± 0.139	
Total	71	100.0	0.369 ± 0.168^g^	

NOTES. Androgen receptor (AR) expression: absent, 0%; poor, ≥1 to 30%; moderate, >30 to 60%; high, >60%. Estrogen receptor (ER)-positive : ER expression ≥10%.

^a^Two women had missing information on ER status. ^b^Test for linear trend. ^c^Fisher’s test. ^d^Linear contrast comparing AR-high to AR-absent, AR-low and AR-moderate combined. ^e^Linear contrast comparing AR-absent to AR-low and AR-moderate combined. ^f^Bonferroni adjusted *P* value. ^g^Fisher’s test *P*=0.023.

Logistic regression analysis further illustrated that ER-negative and ER-positive groups showed different age-adjusted relationships between testosterone and AR expression (Table 3). For women with ER-positive tumors, those in the highest testosterone tertile were significantly more likely to have high AR expression than women in the lowest tertile, with age-adjusted OR of 1.86 (95% CI, 1.10-3.16) (Table 3). This association between high testosterone and high AR expression was essentially limited to women aged more than 65 years, with age-adjusted OR of 2.42 (95% CI, 1.22-4.82). High testosterone was also associated to AR-absent tumors, however not significantly, although the low number in this category may limit the appearance of significant effects: the OR for the highest tertile was 1.92 (95% CI, 0.73–5.02). This association between high testosterone and AR-absent tumors was present in both age groups with similar patterns (Table 3). Other interesting information was derived from analyses on the ER-negative tumors. In women with ER-negative tumors, high testosterone was not related to AR-high expression but only to AR-absent expression (with borderline significance). The age-adjusted OR of having AR-absent instead of AR-positive tumors, comparing the highest to the first and second pooled tertiles (the low number of women required to pool the data), was 2.82 (95% CI, 0.98-8.06) (data not in Tables). The OR within the considered age groups could not be estimated because too few subjects were available, but the association appeared limited to women aged ≥65 years. For older women, the ratio AR-absent/AR-positive tumors was 6/15 in the first and second pooled tertiles and 7/2 in the third testosterone tertile, whereas for younger women the ratio AR-absent/AR-positive tumors was 10/19 in the first and second pooled tertiles and 5/7 in the third testosterone tertile.

**Table 3:** **Odds ratios of AR expression by testosterone tertiles in natural postmenopausal women with ER**-**positive breast cancer**

	**Testosterone tertiles, ng/ml**			
	**≤0.328**	**0.328-0.474**	**> 0.474**	***P t*rend**
All ages				
AR expression				
Absent / poor-moderate	8/70	11/73	12/55	
age-adjusted OR (95% CI)	1	1.31 (0.50-3.46)	1.92 (0.73-5.02)	0.189
High / poor-moderate	42/70	45/73	62/55	
age-adjusted OR (95% CI)	1	1.04 (0.61-1.77)	1.86 (1.10-3.16)	0.020
<65 years				
AR expression				
Absent / poor-moderate	3/27	6/39	5/25	
age-adjusted OR (95% CI)	1	1.38 (0.32-6.05)	2.03 (0.43-9.63)	0.374
High / poor-moderate	18/27	23/39	21/25	
age-adjusted OR (95% CI)	1	0.88 (0.40-1.95)	1.26 (0.55-2.91)	0.582
≥65 years				
AR expression				
Absent / poor-moderate	5/43	5/34	7/30	
age-adjusted OR (95% CI)	1	1.25 (0.33-4.70)	2.00 (0.58-6.90)	0.282
High / poor-moderate	24/43	22/34	41/30	
age-adjusted OR (95% CI)	1	1.13 (0.54-2.37)	2.42 (1.22-4.82)	0.011

NOTES. Androgen receptor (AR) expression: absent, 0%; poor, ≥1 to 30%; moderate, >30 to 60%, high: >60%. Estrogen receptor (ER) -positive: ER expression ≥10%.

## Discussion

In the present study, we explored the role of AR in breast cancer by evaluating relationships between AR expression, serum testosterone levels, and some patient (age, BMI) and tumor characteristics (size, nodal involvement, histology, grade, ER status, PR status, HER2 status) in a cohort of postmenopausal patients. The association of testosterone and AR expression was more evident in patients who experienced natural menopause than in the whole cohort, which included patients with surgical menopause (see Additional file 1, Table S3). We therefore focused our attention on patients in natural menopause. Our main finding was that elevated testosterone levels were associated with AR-highly-positive expression in ER-positive tumors (a highly significant relationship) but, surprisingly, elevated testosterone levels were also associated with AR-absent expression in ER-negative tumors (borderline significance). The strong relationship of testosterone and AR in ER-positive tumors was essentially due to patients ≥65 years, which was responsible for the significant association found in the whole cohort. We also found that AR positivity was significantly related to low histological grade, ER-positive status and PR-positive status and was also associated, although not significantly, to small tumor size (<2 cm). The relationships of AR expression with age, BMI and HER2 status were weaker in naturally menopausal women and more evident in all postmenopausal women; and the association with axillary nodal status was virtually absent in both natural and all women groups (see Additional file 1, Table S1).

AR-positivity (poor, moderate, and high) was present in about 85% of tumors in our cohort, a percentage comparable to that of the other studies [5-10,16], which often regarded as AR-positive only those tumors with more than 10% of stained cells. Relationships of AR positivity with low grade, ER-positive and PR-positive status are well documented [5-10,16], and associations with tumor size and axillary nodal involvement have been reported in some studies [7-9,11] but not in others [8,10].

Our finding that AR are more frequently, although not significantly, expressed in older than in younger postmenopausal patients has also been reported in several other studies [6,10,12]. The association of AR expression with old age fits well with the previous finding on the same cohort that testosterone levels show a slight, not significant, increase in the oldest patients [13]. It is well known that the risk of developing breast cancer increases markedly with advancing age [17,18], and signs of masculinization — markers of enhanced androgenic activity — are often present in aged women [19], suggesting a possible link between the androgen/AR pathway and increased risk of breast cancer in an old age.

Our finding that elevated serum testosterone levels are significantly related with both ER-positivity [13] and AR-positivity suggests that an androgen excess may be not only a marker of hormone-dependence but that it may play a role in the development of these hormone-dependent tumors. The most plausible mechanism by which androgen excess stimulates growth of ER-positive/AR-positive cancers is increased conversion to estrogens, as suggested by the well-documented finding of estradiol concentrations 10 times higher in tumor tissue than in blood [20-27] and by evidence of increased expression of estrogen-producing enzymes in breast cancer tissue [28-35], which is suggestive of local synthesis of estradiol from androgen precursors. Estradiol is therefore the final stimulator of breast epithelium proliferation, in agreement with the widely recognized role of estrogens in breast cancer. Increased expression of the androgen-producing enzyme 5α-reductase is also well recognized in breast cancer tissue [29,32]: 5α-reductase catalyzes the conversion of testosterone into the stronger and non-aromatizable dihydrotestosterone, thus explaining reports of dihydrotestosterone concentrations three times higher in tumor tissue than in blood [24,25]. Finally, testosterone and dihydrotestosterone probably up-regulate intratumor AR synthesis, which would account for the frequent co-existance of ER and AR in the same tumor.

Summing up our reasoning on hormone-dependent breast cancer growth, we suggest that most of the findings reported in the literature, including high intratumor concentrations of androgens and estrogens, elevated expression of estrogen-producing and androgen-producing enzymes, increased expression of ER and AR, can be explained by an androgen excess. Furthermore, looking at breast cancer growth under the viewpoint of the androgen excess, the elevated intratumor levels of androgens and estrogens should be regarded as two different sides of the same endocrine abnormality of the woman with cancer, i.e., an androgen excess, thus bypassing the problem of whether androgens inhibit or stimulate breast cancer growth.

In the present study, we regarded the AR-absent group as the negative group and classified the other tumors as poorly positive, moderately positive and highly positive according to the percentage of stained cells. The AR-absent group included about 13% of the patients in our cohort: it was characterized by high serum testosterone levels, comparable to those found in patients with highly positive-AR expression and substantially higher than those in poorly and in moderately AR-positive groups. About 47% of AR-absent tumors were also ER-negative, representing approximately 6% of the whole cohort. Our finding that elevated testosterone levels were associated with AR-absent expression in ER-negative tumors identified a particular subset of cancers whose growth may be stimulated by androgens. The positive association between testosterone and tumor size remained significant in this group: mean testosterone levels were 0.348±0.176 for tumor size <2 cm and 0.482±0.188 for tumor size ≥2 cm (Fisher’s *P*=0.043). It is noteworthy that in women with ER-negative tumors the association between testosterone levels and AR expression substantially weakened and virtually disappeared when we classified as AR-negative those tumors with ≤10% or with ≤30% of stained cells, respectively. In the AR-absent/ER-negative subset, elevated androgen levels cannot stimulate cancer growth either directly or after conversion into estrogens, but they probably stimulate increased production of some other substance which is responsible for cancer growth through binding to its specific receptor. We suggest that such a substance may be the epidermal growth factor (EGF), whose synthesis and function is under the control of androgens [36] and whose receptor (EGFR or HER1) is expressed in 13-44% of breast cancers [37-41] and in 6% of cases in a study by Barghava et al. [42], who used more stringent criteria in defining EGFR overexpression.

HER2 expression was examined in 70% of the tumors of our cohort. We did not find a significant association between HER2 and AR expression in the whole cohort, but when we divided women by ER status, HER2 overexpression showed a significant inverse relationship with AR-high expression in the ER-positive subset (women with HER2 overexpression were 36.0% in the AR-high group and 52.2% in the lower AR expression group, *P*=0.007). In the ER-negative subset, HER2 overexpression was found significantly associated with AR positivity (women with HER2 overexpression were 82.9% in the AR-positive group and 29.2% in the AR-absent group, *P*<0.001).

ER-negative/AR-positive tumors are regarded as the molecular apocrine subtype described by Farmer et al. [43], and the association between HER2 and AR has been repeatedly reported in these tumors [5,6,43-45]. Naderi et al. [45] demonstrated a functionally significant cross-talk between AR and HER2 in molecular apocrine tumors, whose growth is stimulated by androgens [44,45].

We conclude our discussion with a brief comment about the protective role of androgens in breast cancer that has been postulated by several researchers on the basis of clinical evidence and preclinical studies [46,47]. Clinical evidence includes remission of metastases in 20-30% of patients treated with androgens at high doses; this is about the same remission rate of metastatic disease that is obtained with estrogens at high dosage. Preclinical studies on the role of androgens in breast cancer have been summarized in the review by Liao and Dickson [48]: in animals, androgens were mostly shown to inhibit cancer development and to favor regression of already established tumors in several studies, but in some experiments androgens were shown to enhance tumor growth. The same inconclusive results were obtained in cell-culture studies, in which results were dependent on cell types and experimental conditions.

In healthy postmenopausal women, the totality of estrogens and large amounts of active androgens are synthesized in peripheral tissues from the adrenal precursor dehydroepiandrosterone (DHEA) [47,49-51]. A protective role in breast cancer has been suggested for DHEA and an increased risk of breast cancer has been attributed to the progressive decline in the production of the hormone with advancing age [47,49-51]. It has been calculated that postmenopausal ovaries contribute about 20% of circulating testosterone in healthy women, [47,51], but a much larger contribution may be expected from the ovaries of breast cancer patients. Ovarian androgen secretion is positively associated with the degree of ovarian stromal hyperplasia [Sluijmer et al. and Lucisano et al. as quoted by Labrie et al. 47], and our previous studies showed that interstitial cell hyperplasia is a typical feature of the ovaries of breast cancer patients with elevated testosterone levels [52,53]. Further evidence that the increased testosterone levels were of ovarian origin was provided by the significant reduction of testosterone excretion after oophorectomy [54,55]. As a final comment, the suggestion that an increased risk of breast cancer is associated to the progressive decrease in DHEA levels with age (adrenopause) contrasts with the evidence that high levels of adrenal androgen precursors are present in breast cancer tissue, a finding that implicates increased local production of active androgens and estrogens independently from the circulating levels of DHEA [21,23,56].

## Conclusions

Summing up findings in the present study, we have shown that (a) relationships between AR expression and tumor characteristics are in agreement with reports in the literature; (b) the association with AR expression confirms testosterone levels as a marker of hormone-dependent disease; (c) a subset of patients characterized by AR-absent expression and elevated levels of testosterone has been identified; and (d) the contemporary evaluation of ER status, AR expression, and circulating testosterone levels may identify different subsets of cancers whose growth may be influenced by androgens. These findings provide further support to the androgen excess theory of breast cancer, which points to androgen excess as a stimulatory hormonal alteration common to several breast cancer types, both ER-positive and ER-negative [57]. Breast cancer comprises a heterogeneous group of tumors that differ in clinical behavior, response to therapy, and outcome. Evidence exists that the androgen/AR pathway stimulates the growth of ER-negative/AR-positive tumors and AR-targeted therapy has been proposed for the treatment of these tumors [7,9,58]. Findings of the present study suggest that the evaluation of serum testosterone levels may provide a better characterization of different subsets of breast cancer and may provide additional information on the role of the androgen/AR pathway in the regulation of breast cancer growth.

## Abbreviations

AR: Androgen receptors; BMI: Body mass index; DHEA: Dehydroepiandrosterone; EGF: Epidermal growth factor; EGFR: Human epidermal growth factor receptor; HER1: Human epidermal growth factor receptor 1; HER2: Human epidermal growth factor receptor 2; ER: Estrogen receptor; INT: Fondazione IRCSS Istituto Nazionale dei Tumori; 95% CI: Ninety-five percent confidence intervals; OR: Odds ratio; PR: Progesterone receptor; RIA: Radioimmunoassay; TPM: Testosterone, prognosis, mammary cancer.

## Competing interests

The authors declare that they have no competing interests.

## Authors’ contributions

Giorgio Secreto and Andrea Micheli: conception and design of the study, interpretation of data and drafting of the manuscript. Elisabetta Meneghini: analysis and interpretation of data and drafting of the manuscript. Elisabetta Venturelli: acquisition and interpretation of data, and drafting of the manuscript. Maria Luisa Carcangiu and Biagio Paolini: evaluation of AR expression. Roberto Agresti, Patrizia Cogliati, Giuseppina Saragò, Cristina Pellitteri: acquisition of data. Franco Berrino and Massimo Gion: critical revision of the manuscript. All authors read and approved the final manuscript.

## Pre-publication history

The pre-publication history for this paper can be accessed here:

http://www.biomedcentral.com/1471-2407/12/599/prepub

## Supplementary Material

Additional file 1 **A word file containing tables and relating comments of supplementary analyses on all (natural and surgical) and on surgical postmenopausal women.** Additional Table S1: Androgen receptor (AR) expression and characteristics of all postmenopausal breast cancer women. Additional Table S2: Serum testosterone (mean ± SD) by androgen and estrogen receptor expression in breast cancer women who had had a surgical menopause. Additional Table S3: Odds ratios of AR expression by testosterone tertiles in all postmenopausal women with ER-positive breast cancer. Additional Table S4: Odds ratios of AR expression by testosterone tertiles in postmenopausal women with ER-negative breast cancer (all postmenopausal and naturally menopausal women). Click here for file
