# Supplementary material for: Androgen receptors and serum testosterone levels identify different subsets of postmenopausal breast cancers
Source: BMC Cancer. 2012 Dec 14;12:599. doi: 10.1186/1471-2407-12-599 (PMC3554552; doi:10.1186/1471-2407-12-599)
Supplement: Additional file 1 — A word file containing tables and relating comments of supplementary analyses on all (natural and surgical) and on surgical postmenopausal women. Additional Table S1: Androgen receptor (AR) expression and characteristics of all postmenopausal breast cancer women. Additional Table S2: Serum testosterone (mean ± SD) by androgen and estrogen receptor expression in breast cancer women who had had a surgical menopause. Additional Table S3: Odds ratios of AR expression by testosterone tertiles in all postmenopausal women with ER-positive breast cancer. Additional Table S4: Odds ratios of AR expression by testosterone tertiles in postmenopausal women with ER-negative breast cancer (all postmenopausal and naturally menopausal women). [file 1471-2407-12-599-S1.docx]

**Additional File 1**

In this Additional file 1, we present results on our entire postmenopausal cohort (i.e. including both women with natural and women with surgical menopause), thus making possible a comparison of our data with those of the scientific literature.

In all postmenopausal women, the AR expression distribution was similar to that shown for the natural-menopause women subgroup (cf. Table S1 and Table 1). However, when the association between all the considered variables with AR expression was analyzed, some discrepancies between all postmenopausal women and the natural-menopause women subgroup appeared. Specifically, in comparison to results obtained when studying the natural-menopause subgroup, the following considerations emerged when the present and larger set was studied:

(a) the highest AR expression in the oldest age was slightly more evident, though not yet significantly; (b) tumors with high AR expression were present with a higher frequency (not significantly) in women with BMI ≥ 30 kg/m^2^; (c) the inverse association of high AR expression and large tumor size — ≥ 2 cm — was slightly less evident; (d) the decreasing frequency of infiltrating ductal carcinoma (IDC) across increasing categories of AR expression was more evident and becoming significant (*P* trend = 0.049).

As expected, mean testosterone levels of women who had had a surgical menopause were lower than those of women in natural menopause (cf. Table S2 vs. Table 2). Moreover, their mean testosterone levels did not significantly differ between the AR expression categories.

In the entire menopausal set with ER-positive tumours, the inclusion of women who had had a surgical menopause in the logistic regression somehow modified the results obtained for the natural-menopause analysis. In the large set analysis, a weaker association **was** noted between high testosterone levels with (a) the AR-high expression (mainly for women aged ≥65 years), and (b) the AR-absent expression (mainly for women aged <65 years) (cf. Table S3 vs. Table 3). **In** contrast, in the ER-negative group, the association of high testosterone levels with AR-absent expression became significant, essentially for the increased number of analyzed subjects (Table S4).

Table S1. Androgen receptor (AR) expression and characteristics of postmenopausal breast cancer women

|  | AR expression | | | | | | | |  |
| --- | --- | --- | --- | --- | --- | --- | --- | --- | --- |
|  | absent | | poor | | moderate | | high | |  |
|  | n | % ^a^ | N | % ^a^ | N | % ^a^ | n | % ^a^ | p^b^ |
| Age, years |  |  |  |  |  |  |  |  |  |
| < 65 | 35 | 48.0 | 63 | 48.1 | 64 | 46.7 | 74 | 39.6 | 0.122 |
| ≥ 65 | 38 | 52.0 | 68 | 51.9 | 73 | 53.3 | 113 | 60.4 |  |
| BMI, kg/m^2^ |  |  |  |  |  |  |  |  |  |
| < 25 | 38 | 54.3 | 47 | 43.1 | 64 | 50.8 | 74 | 43.8 | 0.131 |
| 25-30 | 21 | 30.0 | 46 | 42.2 | 41 | 32.5 | 54 | 31.9 |  |
| ≥ 30 | 11 | 15.7 | 16 | 14.7 | 21 | 16.7 | 41 | 24.3 |  |
| Tumor size, cm |  |  |  |  |  |  |  |  |  |
| < 2 | 43 | 59.7 | 87 | 66.9 | 84 | 61.8 | 129 | 70.1 | 0.188 |
| ≥ 2 | 29 | 40.3 | 43 | 33.1 | 52 | 38.2 | 55 | 29.9 |  |
| Histology |  |  |  |  |  |  |  |  |  |
| Infiltrating ductal carcinoma (IDC) | 61 | 88.4 | 104 | 81.3 | 107 | 79.3 | 143 | 76.9 | 0.049 |
| Other infiltrating carcinoma | 8 | 11.6 | 24 | 18.7 | 28 | 20.7 | 43 | 23.1 |  |
| Grade |  |  |  |  |  |  |  |  |  |
| ≤ 2 | 28 | 38.9 | 80 | 61.5 | 83 | 61.9 | 133 | 71.5 | <0.001 |
| > 2 | 44 | 61.1 | 50 | 38.5 | 51 | 38.1 | 53 | 28.5 |  |
| Axillary nodal status |  |  |  |  |  |  |  |  |  |
| Negative | 42 | 60.9 | 76 | 59.4 | 87 | 64.4 | 113 | 61.8 | 0.751 |
| Positive | 27 | 39.1 | 52 | 40.6 | 48 | 35.6 | 70 | 38.2 |  |
| ER status |  |  |  |  |  |  |  |  |  |
| Negative | 36 | 49.3 | 25 | 19.2 | 17 | 12.4 | 12 | 6.5 | <0.001 |
| Positive | 37 | 50.7 | 105 | 80.8 | 120 | 87.6 | 174 | 93.5 |  |
| PR status |  |  |  |  |  |  |  |  |  |
| Negative | 45 | 61.6 | 46 | 35.9 | 41 | 29.9 | 42 | 22.6 | <0.001 |
| Positive | 28 | 38.4 | 82 | 64.1 | 96 | 70.1 | 144 | 77.4 |  |
| HER2 status ^c^ |  |  |  |  |  |  |  |  |  |
| Negative | 35 | 56.5 | 39 | 41.5 | 43 | 45.3 | 73 | 59.8 | 0.211 |
| Positive | 27 | 43.5 | 55 | 58.5 | 52 | 54.7 | 49 | 40.2 |  |
| Total | 73 | 13.8 ^d^ | 131 | 24.8 ^d^ | 137 | 26.0 ^d^ | 187 | 35.4 ^d^ |  |

NOTES. Androgen receptor (AR) expression: absent, 0%; poor, ≥1 to 30%; moderate, >30 to 60%, high: >60%. Estrogen receptor (ER) -positive: ER expression ≥10%.

^a^Column percentage. ^b^Chi-square test for trend. ^c^One hundred fifty-five women (29.4%) had missing information on HER2 status.  ^d^Row percentage.

Table S2. Serum testosterone by androgen and estrogen receptor expression in surgical postmenopausal women with breast cancer

|  | Testosterone | | | |
| --- | --- | --- | --- | --- |
|  | n | % | Mean ± SD (ng/ml) | p |
| Surgical postmenopausal women | |  |  |  |
| AR expression |  |  |  |  |
| absent | 13 | 17.6 | 0.339 ± 0.139 | 0.384 ^b^  0.512 ^c^ |
| poor | 19 | 25.6 | 0.396 ± 0.195 |  |
| moderate | 15 | 20.3 | 0.324 ± 0.144 |  |
| high | 27 | 36.5 | 0.326 ± 0.195 |  |
| Total ^a^ | 74 | 100.0 | 0.346 ± 0.176 |  |
| ER-positive |  |  |  |  |
| AR expression |  |  |  |  |
| absent | 6 | 10.7 | 0.311 ± 0.093 | 0.364 ^b^  0.370 ^d^ |
| poor | 14 | 25.0 | 0.423 ± 0.153 |  |
| moderate | 11 | 19.6 | 0.355 ± 0.146 |  |
| high | 25 | 44.7 | 0.331 ± 0.202 |  |
| Total | 56 | 100.0 | 0.357 ± 0.172 ^e^ |  |
| ER-negative |  |  |  |  |
| AR expression |  |  |  |  |
| absent | 7 | 38.9 | 0.362 ± 0.173 | 0.343 ^b^  0.805 ^c^ |
| poor | 5^f^ | 27.8 | 0.321 ± 0.290 |  |
| moderate | 4^g^ | 22.2 | 0.238 ± 0.111 |  |
| high | 2^h^ | 11.1 | 0.263 ± 0.061 |  |
| Total | 18 | 100.0 | 0.312 ± 0.188 ^e^ |  |

NOTES. AR expression: absent, 0%; poor, ≥1 to 30%; moderate, >30 to 60%, high: >60%. ER-negative: <10%; ER-positive≥10%.

^a^Two women had missing information on ER status. ^b^Test for linear trend. ^c^Fisher’s test. ^d^Kruskal-Wallis test (criterion of homoscedasticity not met). ^e^Fisher’s test p = 0.361.
